# Food readjustment plus exercise training improves cardiovascular autonomic control and baroreflex sensitivity in high‐fat diet‐fed ovariectomized mice

**DOI:** 10.14814/phy2.15609

**Published:** 2023-03-10

**Authors:** Bruno Nascimento‐Carvalho, Adriano Dos‐Santos, Nicolas Da Costa‐Santos, Sabrina L. Carvalho, Oscar A. de Moraes, Camila P. Santos, Katia De Angelis, Erico C. Caperuto, Maria‐Claudia Irigoyen, Katia B. Scapini, Iris C. Sanches

**Affiliations:** ^1^ Unidade de Hipertensao, Instituto do Coracao, Hospital das Clinicas, Faculdade de Medicina Universidade de Sao Paulo (InCor‐HCFMUSP) São Paulo Brazil; ^2^ Human Movement Lab São Judas Tadeu University (USJT) São Paulo Brazil; ^3^ Department of Medicine Federal University of São Paulo (Unifesp) São Paulo Brazil

**Keywords:** autonomic nervous system, food adjustment, inflammation, menopause, physical training

## Abstract

Despite consensus on the benefits of food readjustment and/or moderate‐intensity continuous exercise in the treatment of cardiometabolic risk factors, there is little evidence of the association between these two cardiovascular risk management strategies after menopause. Thus, the objective of this study was to evaluate the effects of food readjustment and/or exercise training on metabolic, hemodynamic, autonomic, and inflammatory parameters in a model of loss of ovarian function with diet‐induced obesity. Forty C57BL/6J ovariectomized mice were divided into the following groups: high‐fat diet‐fed ‐ 60% lipids throughout the protocol (HF), food readjustment ‐ 60% lipids for 5 weeks, readjusted to 10% for the next 5 weeks (FR), high‐fat diet‐fed undergoing moderate‐intensity exercise training (HFT), and food readjustment associated with moderate‐intensity exercise training (FRT). Blood glucose evaluations and oral glucose tolerance tests were performed. Blood pressure was assessed by direct intra‐arterial measurement. Baroreflex sensitivity was tested using heart rate phenylephrine and sodium nitroprusside induced blood pressure changes. Cardiovascular autonomic modulation was evaluated in time and frequency domains. Inflammatory profile was evaluated by IL‐6, IL‐10 cytokines, and TNF‐alpha measurements. Only the exercise training associated with food readjustment strategy induced improved functional capacity, body composition, metabolic parameters, inflammatory profile, and resting bradycardia, while positively changing cardiovascular autonomic modulation and increasing baroreflex sensitivity. Our findings demonstrate that the association of these strategies seems to be effective in the management of cardiometabolic risk in a model of loss of ovarian function with diet‐induced obesity.

## Graphical abstract

The objective of this study was to evaluate the effects of food readjustment and/or exercise training on metabolic, hemodynamic, autonomic, and inflammatory parameters in a model of loss of ovarian function with diet‐induced obesity. The findings of the present study lend support to the hypothesis that exercise training and food readjustment promote specific benefits in some of the evaluated parameters. However, only the association of food readjustment with exercise training was effective in promoting metabolic, hemodynamic, autonomic, and inflammatory benefits in a model of loss of ovarian function with diet‐induced obesity.

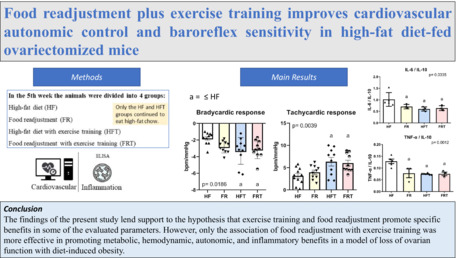

## 1 INTRODUCTION

Menopause is an aging‐related natural event, characterized by the final menstrual period with the loss of ovarian function and progressive decline in endogenous estrogen levels (Ambikairajah et al., 2019; Pae et al., 2018). It is well‐established that the hormonal changes in menopause are associated with an increase in central adiposity and changes in body fat distribution (Ambikairajah et al., 2019; Peppa et al., 2012). Experimentally, ovariectomy is an established model for mimicking the changes in human menopause, because it has several similar human risk factors, such as increased blood pressure, impaired myocardial function and autonomic modulation, as well as reduced baroreflex sensitivity (Braga et al., 2015; Sanches et al., 2015; Shimojo et al., 2018).

The association of menopause with changes in adipose tissue also suggests metabolic damage, in the production of pro‐inflammatory cytokines, increasing free fatty acids and visfatin production. These adaptations are probably associated with an increase in susceptibility to insulin resistance, dyslipidemia, and hypertension (Peppa et al., 2012). Indeed, the loss of ovarian function added to a diet‐induced obesity increased metabolic risks, body weight, and adiposity, along with inflammatory profile damage (Ludgero‐Correia et al., 2012; Pae et al., 2018).

It has been reported that the reduction of ovarian hormones is related to sympathovagal imbalance associated with reduced parasympathetic modulation (Adachi et al., 2011). In addition, when obesity is diagnosed, changes, such as baroreflex impairment, increase in leptin, insulin resistance, and obstructive sleep apnea, are all indicative of increased sympathetic nervous system activity, probably associated with a down‐regulation of beta‐adrenergic receptors (Guarino et al., 2017). These changes may lead to increased blood pressure and susceptibility to hypertension (Guarino et al., 2017). Indeed, obesity in postmenopausal women poses major cardiovascular risks, with elevated systolic and diastolic blood pressure associated with high arterial stiffness (Son et al., 2017).

The guidelines of the American Heart Association (AHA), the American College of Cardiology (ACC), among others, recommend changes in dietary patterns, increased level of physical activity, and reduced body weight (Whelton et al., 2018). Indeed, food readjustment is effective in promoting increased vagal modulation in severe obesity (Paul et al., 2003). Increasing vagal modulation and decreasing sympathovagal balance in a population with obesity‐associated type 2 diabetes may induce resting bradycardia (Ziegler et al., 2015). Moreover, food readjustment can contribute to reducing systolic blood pressure in normal and ovariectomized rats (Roberts et al., 2001).

In parallel with dietary effects, the literature reports positive effects of physical training on baroreflex sensitivity, decrease in oxidative stress, resting bradycardia and parasympathetic modulation in experimental models of loss of ovarian function (da Palma et al., 2016; Irigoyen et al., 2005). This is associated with hemodynamic, autonomic, and inflammatory benefits in a model of metabolic syndrome with loss of ovarian function (Conti et al., 2015).

Although both types of intervention promote improvements, each change different parameters. Thus, we should assess the effectiveness of these strategies (food readjustment and/or exercise training) in a condition of obesity plus ovarian deprivation and determine which mechanisms are changed when this nonpharmacologic approach is adopted. Thus, the objective of this study was to evaluate the effects of food readjustment and/or exercise training on metabolic, hemodynamic, autonomic, and inflammatory parameters in a model of loss of ovarian function with diet‐induced obesity. Therefore, our hypothesis is that the deleterious cardiovascular, metabolic, and inflammatory effects due ovarian hormones deprivation and high‐fat diet may be attenuated by physical training and food readjustment.

## 2 METHODS

### 2.1 Experimental model and study groups

**FIGURE 1:**
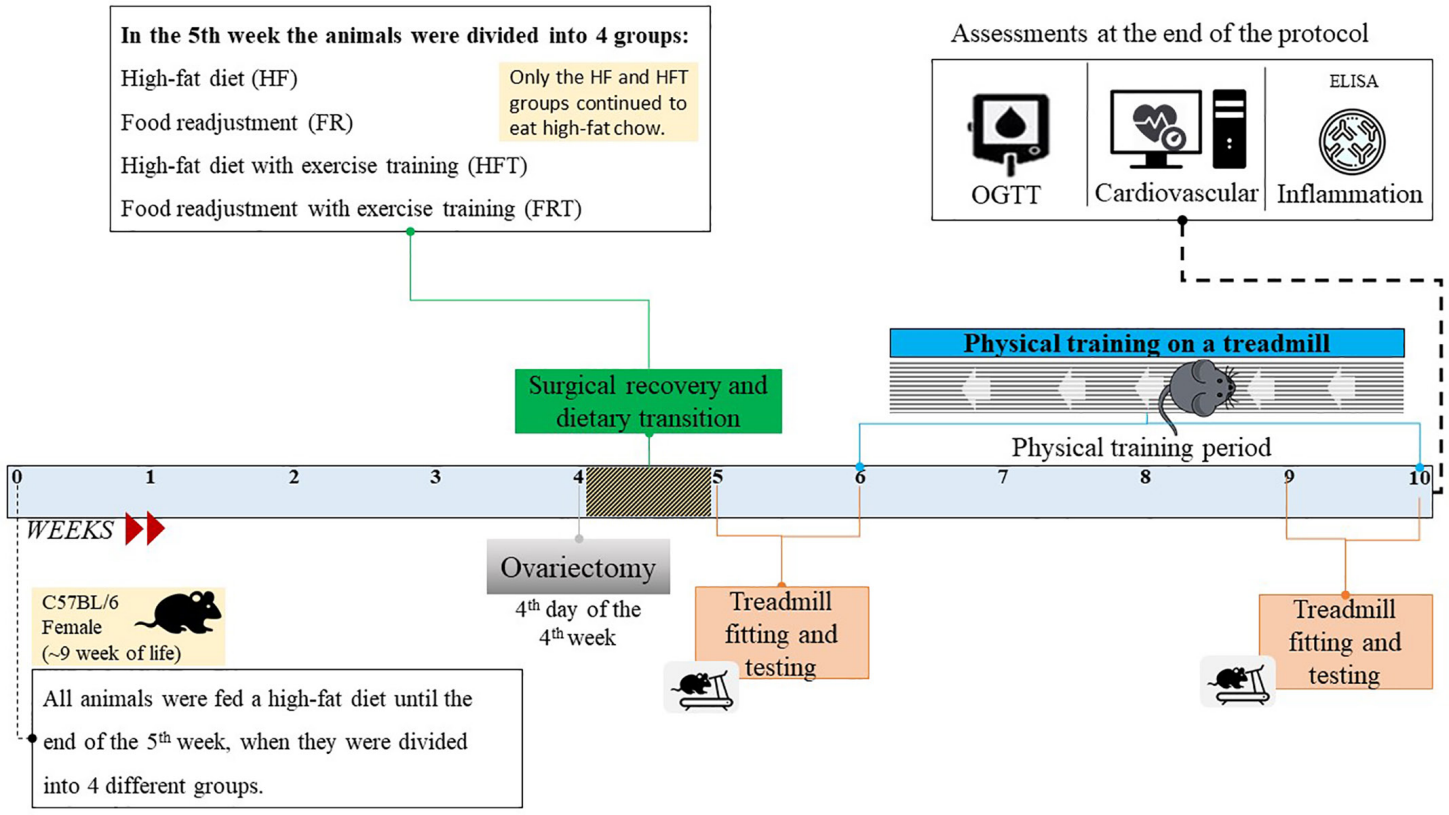
Protocol diagram.

Experiments were performed using 40 female C57BL/6J mice, 9 weeks of age, from the School of Medicine of the University of São Paulo, housed in a temperature‐controlled room (22°C) with a 12‐h dark/light cycle. Until the 5th week of the protocol all animals had the same treatment. They were fed a high‐fat diet and after the recovery of ovariectomy surgery they were reallocated into each of the 4 experimental groups. They are:

1. HIGH‐FAT (HF)—fed a high‐fat diet (60% lipids) until the end of the protocol;
2. FOOD READJUSTMENT (FR)—fed a low‐fat diet (10% lipids) until the end of the protocol;
3. HIGH‐FAT DIET WITH EXERCISE TRAINING (HFT)—fed a high‐fat diet until the end of the protocol, plus exercise training from the 6th to 10th weeks of protocol;
4. FOOD READJUSTMENT WITH EXERCISE TRAINING (FRT)—fed a low‐fat diet until the end of the protocol, plus exercise training from the 6th to 10th protocol week;

The mice received Ain‐93 (chow with 10% lipids) or Ain‐93 adapted (chow with 60% lipids) (Reeves et al., 1993). Food readjustment was characterized by consumption of a high‐fat diet (Ain‐93 adapted) during 5 weeks, followed by consumption of feed (Ain‐93) until the end of the protocol (5 weeks). The high‐fat diet groups consumption of chow with 60% lipids continued for the entire protocol (10 weeks), The exchange of diet mentioned above aimed to verify the influence of dietary adjustment alone or associated with the practice of physical training. All animals were anesthetized (50 mg/kg ketamine and 10 mg/kg xylazine, intraperitoneal, i.p), and a small abdominal incision was performed, the oviduct was sectioned, and the ovaries removed (Heeren et al., 2009; Marchon et al., 2015). To clarify the moments of the experiment, a protocol diagram is presented in Figure 1. The Ethics Committee of Sao Judas Tadeu has approved the research project (protocol number 025/2016).

The animals had to walk and run on a motorized treadmill (10 min/day; 0.3 km/h) for 4 consecutive days, before the maximal running test. The maximal running test was performed by all groups as described in detail in a previous study (De Angelis et al., 2004; Heeren et al., 2009). Exercise training was performed on a treadmill (Imbramed TK‐01, Brazil) at moderate intensity (∼60%–80% maximal running speed) for 1 h per day, for 4 weeks (Heeren et al., 2009; Marchon et al., 2015).

At the end of the experiment, gastrocnemius and white adipose tissue were collected after euthanasia (Flues et al., 2010). All procedures were approved by the Institutional Ethics Committee of São Judas University (025/2016), according to the guidelines of the National Council of Control of Animal Experimentation.

### 2.2 Measurement of blood glucose level and Oral glucose tolerance test (OGTT)

The animals were fasted overnight for 12 h (8 pm–8 am) with free access to water. Glucose was measured using an Accu‐Check Advantage Blood Glucose Monitor (Roche Diagnostic Corporation). Animals were fasted for 12 h, given a gavage glucose load (1.4 g/kg), and blood samples were taken at baseline and 15, 30, 60, 90, and 120 min from a cut made at the tip of the tail (Song et al., 2017).

### 2.3 Hemodynamic measurements

Two days after the last training session, mice were anesthetized (mixture of 0.5%–2% isoflurane and 98% O_2_ at a flow rate of 1.5 L/min), and polyethylene‐tipped Tygon cannulas filled with heparinized saline were inserted into the carotid artery and jugular vein for direct measurements of arterial pressure (AP) and drug administration, respectively.

Two days after surgery, hemodynamic measurements were performed, the animals were awake and allowed to move freely in their cages. The cannula was coupled to a biological signal transducer for recording blood pressure signals (Blood Pressure XDCR, Kent Scientific) for 30 min using a digital converter (Windaq DI720, 4‐kHz sampling frequency, Dataq Instruments) (Heeren et al., 2009; Marchon et al., 2015). The recorded data were analyzed on a beat‐to‐beat basis to quantify changes in mean arterial pressure (MAP) and HR.

### 2.4 Baroreflex sensitivity evaluation

Baroreflex sensitivity was evaluated by tachycardic or bradycardic responses induced by two injections of sodium nitroprusside (8 g/kg body wt IV) or phenylephrine (8 g/kg body wt IV), respectively. Data were expressed as beats per minute (bpm) per mm Hg. Maximal dose per injection was <20 μg (De Angelis et al., 2004).

Peak increases or decreases in MAP after phenylephrine or sodium nitroprusside injection and the corresponding peak reflex changes in HR were recorded for each drug dose. The drugs were administered randomly in all animals, its response peaks (maximum blood pressure change) were usually observed between 3 to 4 s with phenylephrine, and response peaks were usually observed between 8 and 10 s with sodium nitroprusside. After the return of blood pressure the baseline the injections were applied. Baroreflex sensitivity was calculated by the ratio between changes in HR to the changes in MAP, allowing a separate analysis of reflex bradycardia and reflex tachycardia.

### 2.5 Cardiovascular autonomic modulation

Pulse interval (PI) variability and systolic arterial pressure (SAP) variability were assessed in time and frequency domains by spectral analysis using Cardioseries *Software* (V2.7). For frequency domain analysis of cardiovascular autonomic modulation, PI and SAP were divided into segments and overlapped by 50%, cubic spline‐decimated to be equally spaced in time after linear trend removal; power spectral density was obtained through the fast Fourier transformation. The components of spectral analysis were quantified in the low‐frequency ranges (LF, 0.4–1.5 Hz) (Pelat et al., 2003; Thireau et al., 2008) and high‐frequency ranges (HF, 1.5–5.0 Hz) (Pelat et al., 2003; Thireau et al., 2008).

### 2.6 Inflammatory mediators

Interleukin 6 (IL‐6), interleukin 10 (IL‐10), and tumor necrosis factor alpha (TNF‐α) levels were determined in adipose tissue using a commercially available ELISA kit (R&D Systems Inc.), according to the manufacturer's instructions. ELISA was performed in a 96‐well polystyrene microplate with a specific monoclonal antibody coating. Absorbance was measured at 540 nm in a microplate reader (Shimojo et al., 2018). Moreover, the ratio of pro‐inflammatory cytokines to anti‐inflammatory was performed to analyze the inflammatory profile (Feriani et al., 2018).

### 2.7 Statistical analysis

The data were evaluated in Graph Pad Prism (V 8.0.1). The results are presented as mean ± SEM. Data homogeneity was tested through the Kolmogorov–Smirnov test. Two experimental groups were compared using one‐way ANOVA with Tukey post hoc test. The significance level adopted was *p* < 0.05.

## 3 RESULTS

Body composition (final body weight, gastrocnemius, and white adipose tissue), metabolic assessments (blood glucose level and OGTT), the maximal running test (initial and final), and average consumption (grams and calories) are shown in Table 1. The average consumption was increased in relation to the grams in food readjustment groups (HF and HFT vs. FR and FRT). Only the association of food readjustment with exercise training (FRT) reduced body weight and white adipose tissue compared with the control group (HF). Blood glucose level was reduced, and the glucose mobilization capacity was improved represented by the smaller area under the curve in the FRT in relation to high‐fat groups (HF and HFT). Moreover, the trained groups had increased maximum running capacity (HF vs. HFT and FRT).

**TABLE 1:** Average consumption, running capacity, body composition, and metabolic parameters

Parameters	HF	FR	HFT	FRT	*p*
Average consumption of grams (every 2 days)	3.47 ± 018	5.08 ± 0.31^a^	3.23 ± 0.13^b^	5.44 ± 023^a^, ^c^	<0.001
Average consumption of calories (every 2 days)	18.57 ± 0.97	19.31 ± 1.19	17.32 ± 0.68	20.68 ± 0.88	0.115
Maximal running capacity initial (s)	718.30 ± 29.39	692.60 ± 55.39	640.90 ± 31.93	686.50 ± 20.77	0.463
Maximal running capacity final (s)	677.9 ± 50.06	676.9 ± 72.7	929.3 ± 39^ab^	903.9 ± 54.41^a^, ^b^	0.001
Final body weight, (g), 10 weeks	29.1 ± 1.321	27.03 ± 0.7251	27.98 ± 0.8722	22.73 ± 0.5687^abc^	<0.001
Total white of adipose tissue, (g)	0.07768 ± 0.0094	0.05914 ± 0.0058	0.07886 ± 0.0104	0.04942 ± 0.0039^a^	0.032
Blood glucose (mg/dL)	160.1 ± 10.79	131.6 ± 10.1	162.2 ± 7.13	113.1 ± 11.81^a^, ^c^	0.004
OGTT (AUC)	23,137 ± 1223	20,064 ± 703.1	23,722 ± 1144	17,344 ± 1304^a^, ^c^	0.001

*Note*: Different letter indicates statistically different groups (one‐way ANOVA + Tukey test, *p* < 0.05). Data are reported as mean ± SEM. *n* = 10 animal/group. Abbreviations: FR, food readjustment; FRT, food readjustment plus exercise training; HF, high fat; HFT, high fat plus exercise training.

^a^ *p* < 0.05 vs. HF.

^b^ *p* < 0.05 vs. FR.

^c^ *p* < 0.05 vs. HFT.

**FIGURE 2:**
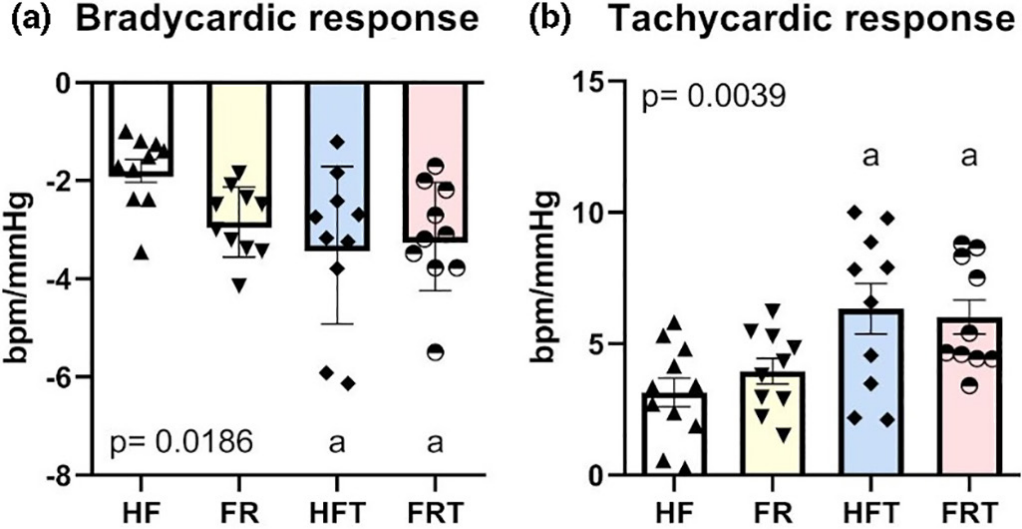
Baroreflex sensitivity parameters in high‐fat (HF) (*n* = 10), food readjustment (FR) (*n* = 10), high‐fat plus exercise training (HFT) (*n* = 10), and food‐readjustment plus exercise training (FRT) (*n* = 10). (a) Bradycardic response; (b) Tachycardic response. Different letter indicates statistically different groups (one‐way ANOVA + Tukey test, *p* < 0.05). Values reported as mean ± SEM. a = *p* < 0.05 vs. HF.

No differences were observed in blood pressure, and resting bradycardia was obtained only by exercise training plus food readjustment (HF vs. FRT) (Table 1). However, baroreflex sensitivity was increased in both trained groups (HFT and FRT) compared with the control group (HF), for bradycardic response (BR: HF: 2.00 ± 0.29; FR: 2.84 ± 0.23; HFT: 3.48 ± 0.54; FRT: 3.46 ± 0.35; bpm/mm Hg, *p* = 0.0155) and tachycardic response (TR: HF: 3.15 ± 0.55; FR: 3.95 ± 0.49; HFT: 6.33 ± 0.96; FRT: 6.02 ± 0.65; bpm/mm Hg, *p* = 0.0039) (Figure 2).

Interestingly, we observed an increase in all parameters of time domain measurements of heart rate variability by exercise training plus food readjustment (HF vs. FRT), the cardiac autonomic modulation (VAR‐PI) and the cardiac parasympathetic modulation (RMSSD) (Table 2). Regarding the time‐domain of cardiac autonomic modulation, no changes were observed in LF‐PI and HF‐PI; however, the LF/HF ratio was reduced only in the exercise training group (HF vs. HFT) (Table 2).

**TABLE 2:** Hemodynamic and heart rate variability evaluations

Parameters	HF	FR	HFT	FRT	*p*
Heart rate	681 ± 13	663 ± 18	627 ± 39	607 ± 14^a^	0.049
(bpm)					
Systolic arterial pressure	134.5 ± 2.60	130.4 ± 2.86	128 ± 3.20	123.6 ± 4.91	0.201
(mm Hg)					
Diastolic arterial pressure	95.09 ± 2.37	94.41 ± 1.89	87.54 ± 5.55	88.3 ± 3.68	0.345
(mm Hg)					
Mean arterial pressure (mm Hg)	115.9 ± 2.15	112 ± 2.15	106.8 ± 4.91	109.2 ± 2.15	0.281
VAR‐PI (ms^2^)	15.67 ± 3.32	49.22 ± 17.11	29.52 ± 10.40	66.98 ± 11.84^a^	0.046
RMSSD (ms)	2.14 ± 0.27	3.01 ± 0.64	2.92 ± 0.59	4.77 ± 0.80^a^	0.049
LF‐PI (ms^2^)	6.03 ± 2.24	1.64 ± 0.64	1.99 ± 0.95	3.59 ± 1.73	0.227
HF‐PI (ms^2^)	1.71 ± 0.37	2.24 ± 1.55	3.15 ± 1.39	3.01 ± 1.69	0.291
LF/HF)	2.83 ±0.61	1.36 ±0.31	0.90 ±0.32^a^	1.22 ±0.31	0.016

*Note*: Different letters indicate statistically different groups (one‐way ANOVA + Tukey test, *p* < 0.05). Data are reported as mean ± SEM. *n* = 10 animal/group. Abbreviations: FR, food readjustment; FRT, food readjustment plus exercise training; HF, high fat; HFT, high fat plus exercise training.

^a^ *p* < 0.05 vs. HF.

**FIGURE 3:**
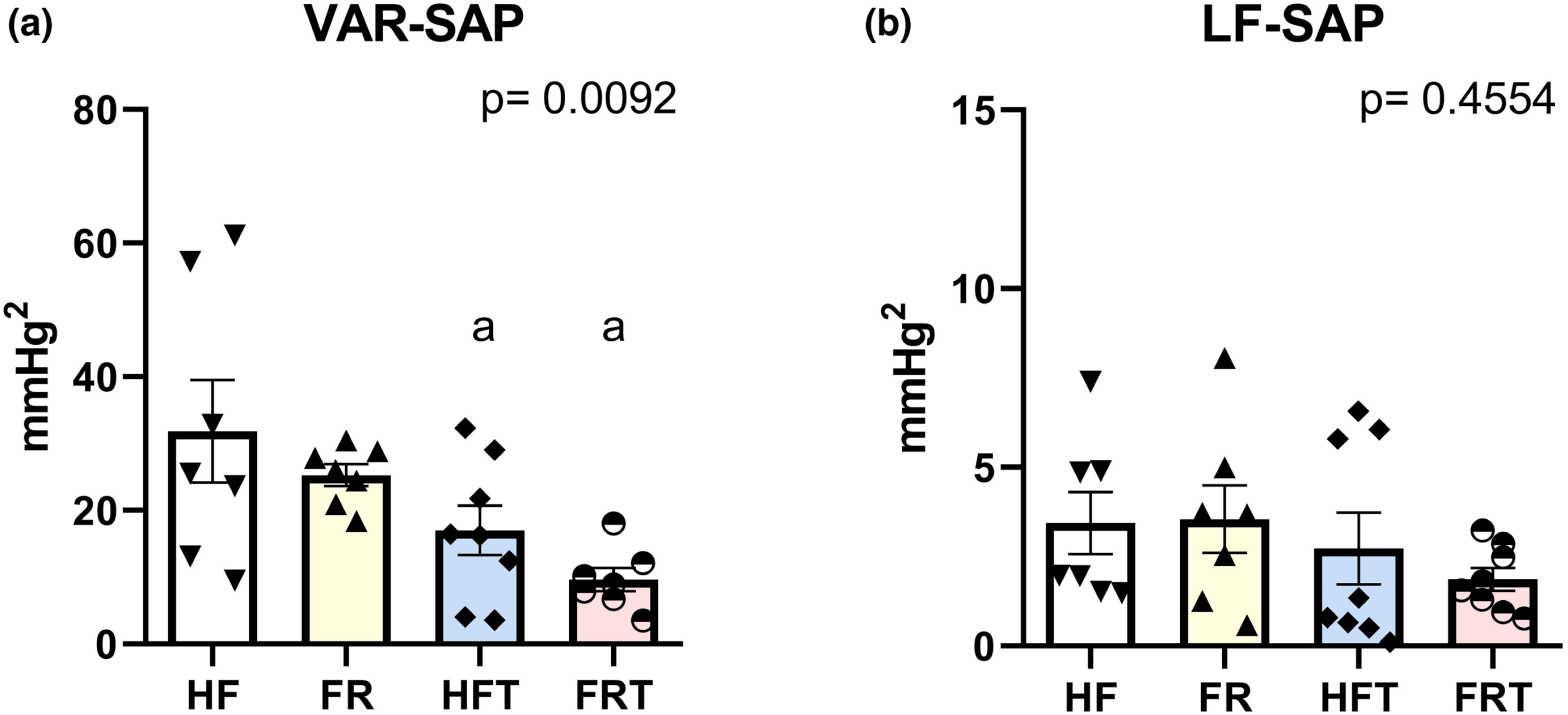
Variability of systolic arterial pressure parameters in high‐fat (HF) (*n* = 7), food‐readjustment (FR) (*n* = 7), high‐fat plus exercise training (HFT) (*n* = 8), and food‐readjustment plus exercise training (FRT) (*n* = 8). (a) Variance of systolic blood pressure (VAR‐SAP); (b) Low‐frequency band of systolic blood pressure (LF‐SAP). Different letter indicates statistically different groups (one‐way ANOVA + Tukey test, *p* < 0.05). Values reported as mean ± SEM. a = *p* < 0.05 vs. HF. b = *p* < 0.05 vs. FR.

The variance of systolic blood pressure was reduced by exercise training plus food readjustment compared with that in the control group (Var‐SAP: HF: 35.59 ± 7.91; FR: 25.25 ± 1.64; HFT: 16.98 ± 3.71; FRT: 9.61 ± 1.74; mm Hg^2^, *p* = 0.0018). However, the low‐frequency band of systolic blood pressure did not change (Figure 3).

**FIGURE 4:**
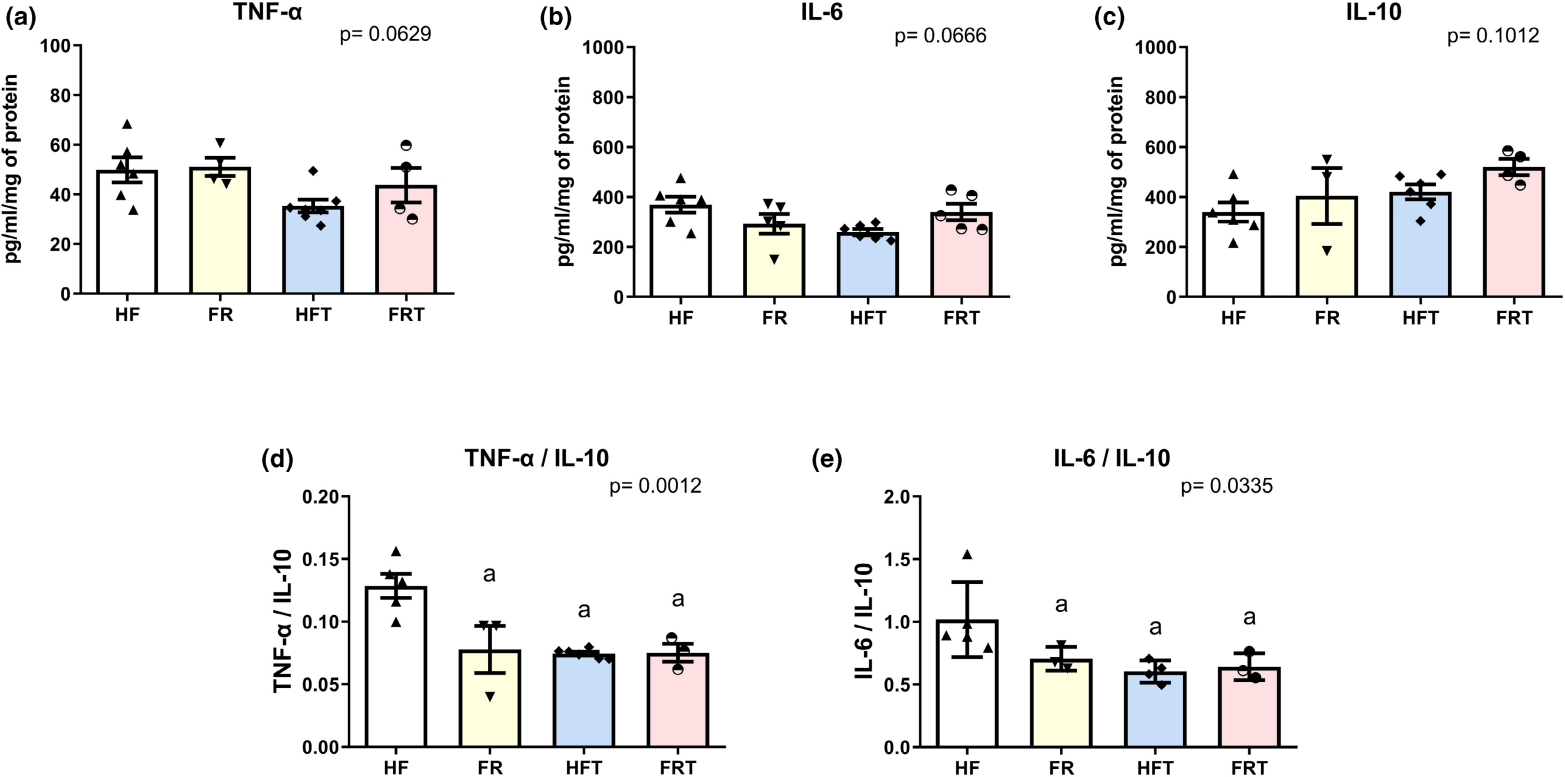
Inflammatory profile in high‐fat (HF) (*n* = 6), food‐readjustment (FR) (*n* = 5), high‐fat plus exercise training (HFT) (*n* = 6), food‐readjustment plus exercise training (FRT) (*n* = 5). (a) TNF‐α; (b) Interleukin‐6 (IL‐6); (c) Interleukin‐10 (IL‐10); (d) ratio of TNF‐ α and Interleukin‐10; (e) ratio of Interleukin‐6 and Interleukin‐10. Different letter indicates statistically different groups (one‐way ANOVA + Tukey test, *p* < 0.05). Values reported as mean ± SEM. a = *p* < 0.05 vs. HF.

No differences were observed in either pro‐inflammatory (IL‐6 and TNF‐alfa) or anti‐inflammatory (IL‐10) parameters (Figure 4). However, when pro‐inflammatory cytokines were associated with anti‐inflammatory ones (IL‐6/IL10 and TNF‐alfa/IL‐10), a reduction was obtained with both interventions (exercise training and/or food readjustment) (IL‐6/IL10: HF: 1.02 ± 0.13; FR: 0.71 ± 0.05; HFT: 0.60 ± 0.04; FRT: 0.64 ± 0.06; pg/mL/mg, *P* = 0.034; TNF‐alfa/IL‐10: HF: 0.13 ± 0.01; FR: 0.08 ± 0.01; HFT: 0.07 ± 0.01; FRT: 0.08 ± 0.01; pg/mL/mg, *p* = 0.001) (Figure 4).

## 4 DISCUSSION

The current study attempted to determine the effects of food readjustment and/or exercise training on metabolic, hemodynamic, autonomic, and inflammatory parameters in a model of loss of ovarian function with diet‐induced obesity. Food readjustment promoted a reduction in pro‐ and anti‐inflammatory cytokine ratio, whereas exercise training also promoted inflammatory profile benefits, increased the maximal running capacity, improved sympathovagal balance, and increased baroreflex sensitivity.

Our findings demonstrated that exercise training plus food readjustment may be a powerful strategy to improve maximal running capacity; body composition was also improved with the decrease in adipose tissue and body weight, leading to changes in metabolic assessments, such as a reduction in blood glucose and OGTT. Resting bradycardia and positive changes in autonomic modulation were observed in the increased heart rate variability, augmented cardiac parasympathetic modulation and reduced vascular sympathetic modulation, increased baroreflex sensitivity, as well as reduced pro‐ and anti‐inflammatory cytokine ratios.

Despite the average increase in the intake of grams in the food readjustment groups, the total intake of calories was equivalent among all groups, which demonstrates that the nature of the macronutrients is probably related to metabolic damage and suggests that leptin action remains unchanged among groups. Studies have shown that with food readjustment the decrease in adipose tissue is accompanied by improvement in glucose metabolization associated with decreased basal glucose (Ziegler et al., 2015). These adaptations in metabolism were obtained only when food readjustment was associated with exercise training. Changes on HRV parameters (SDNN and RMSSD) are accompanied by inversed changes to blood glucose levels (Ernst, 2017). Thus, the better cardiac autonomic control in FRT may have potentialized the expected effect of the food readjustment in glycemic control.

Although the evidence points to a role of nonpharmacologic strategies for the management of high blood pressure, with a lowering of blood pressure by reducing of body weight (Whelton et al., 2018), our findings demonstrate that the reduction in body weight and adipose tissue in this model was not effective in promoting changes in arterial pressure. On the contrary, the association of food readjustment plus exercise training was effective in decreasing vascular sympathetic modulation. A recent meta‐analysis using nonpharmacological approaches for reducing body weight (food readjustment and/or exercise training) seems to lend support to the finding of reduction in sympathetic nervous activity (Costa et al., 2018). As autonomic changes precede other changes (Bernardes et al., 2018), two strategies may be recommended to obtain blood pressure reduction: a longer intervention time or a greater total volume of exercises in a shorter time.

According to the literature, estrogen exhibits vasodilatory properties, and the intracellular transmembrane G protein‐coupled estrogen receptor is one of the vascular estrogen binding sites, next to ERα and ERβ, which is closely related to pressure reduction, in addition to acting in body weight maintenance (Haas et al., 2009). Thus, ovarian deprivation causes increased body weight and blood pressure, and this seems to be linked to the absence of estrogen for its receptors, causing them to lose them functions (Shi et al., 2013).

Resting bradycardia is a regular finding in chronic exercise, generated by an improvement in frank‐starling mechanism and in cardiac autonomic modulation (Almeida & Araújo, 2003; Shimojo et al., 2018). The exercise training‐induced reduction in sympathovagal balance represents a better relationship between the autonomic sympathetic and parasympathetic loops. However, other benefits in cardiac autonomic modulation (increased Var‐PI) and in parasympathetic autonomic modulation (increased RMSSD) were induced by only exercise training plus food readjustment. In fact, in different populations these strategies are effective in promoting this benefit (da Palma et al., 2016; Ziegler et al., 2015).

There is few evidence of cardiac function and histology of the heart in the model used in this study (ovarian deprived female mice, fed high‐fat diet and exercise training). However, it is known that the high‐fat plus ovarian deprivation modifies cardiomyocyte diameter in Wistars rats, promoting cardiac hypertrophy (Goncalves et al., 2017). Additionally, in APOB‐100 transgenic female mice (model of metabolic syndrome) plus consumption of high‐fat diet, exercise training is protective against the development of pathological cardiac hypertrophy, with the maintenance of important indicators of cardiac function (Tóth et al., 2022). Thus, possibly the cardiac structure of mice with ovarian deprivation plus consumption of high‐fat diet presents a series of deleterious adaptations, and exercise training probably can attenuate this picture. However, specific studies are needed to understand these phenomena.

Exercise training improves functional capacity and, consequently, improves baroreflex sensitivity in different types of comorbidities associated with loss of ovarian function (Irigoyen et al., 2005; Shimojo et al., 2018). Indeed, arterial baroreflex is effective in mediating cardiac disorders associated with arterial hypertension, which is crucial for cardiovascular, morpho functional, and autonomic adaptive benefits induced by chronic exercise (Moraes‐Silva et al., 2010). Thus, we can consider that the beneficial effects observed in trained groups in cardiovascular autonomic parameters are associated with the improvement in baroreflex activity in these groups.

In the food readjustment group, the reduction in the inflammatory profile is justified by the shorter period of consumption of a high‐fat diet, and consequently, these animals had a reduction in adipose tissue (↓23.86%) and in inflammation. In addition, a reduction was also expected in the group with the association of strategies.

There were some limitations to this study. First, the absence of a control group that could provide additional answers about the effects to the food readjustment and exercise in without ovariectomy animals. Second, the short period of surgical recovery after the canulation may influence autonomic and hemodynamic outcomes.

### 4.1 Conclusion

The findings of the present study lend support to the hypothesis that exercise training and food readjustment promote specific benefits in some of the evaluated parameters. However, only the association of food readjustment with exercise training was more effective in promoting metabolic, hemodynamic, autonomic, and inflammatory benefits in a model of loss of ovarian function with diet‐induced obesity.

## AUTHORS' CONTRIBUTIONS

Bruno Nascimento‐Carvalho conceived and designed the research, conducted experiments, analyzed data, interpreted results of experiments, drafted the manuscript, edited and revised the manuscript; Adriano dos‐Santos conducted experiments and revised the manuscript; Nicolas Da Costa‐Santos conducted experiments and revised the manuscript; Sabrina L. Carvalho conducted experiments and revised the manuscript; Oscar A. de Moraes analyzed data and revised the manuscript; Camila P. Santos conducted experiments, analyzed data, and revised the manuscript; Katia De Angelis interpreted results of experiments and revised the manuscript; Erico C. Caperuto edited and revised the manuscript; Maria‐Claudia Irigoyen drafted the manuscript, edited and revised the manuscript; Katia B. Scapini interpreted results of experiments and revised the manuscript; Iris C. Sanches conceived and designed the research, interpreted results of experiments, edited and revised the manuscript.

## FUNDING INFORMATION

This study was financed in part by the Coordenação de Aperfeiçoamento de Pessoal de Nível Superior—Brazil (CAPES)—Finance Code 001″; Conselho Nacional de Desenvolvimento Científico e Tecnológico (PQ ‐ CNPq ‐ process 307138/2015–1 and process 435123/2018–1); and ANIMA INSTITUTE—AI. CAPES provided support for personal demands of the main author; CNPq provided support for laboratory demands of research. ANIMA provided support for Human Movement Lab, São Judas University.

## CONFLICT OF INTEREST

No conflicts of interest, financial or otherwise, are declared by the authors.
